# Mortuary Statistics and Meteorological Report

**Published:** 1880-05

**Authors:** 


### APRIL REPORT OF DEATHS AND INTERMENTS IN BALTIMORE.

| CAUSE OF DEATH. | NO. | CAUSE OF DEATH. | NO. | CAUSE OF DEATH. | NO. |
|---|---|---|---|---|---|
| Abscess, Ear | 1 | Disease of Heart | 25 | Malformation | 1 |
| " Peritoneal | 1 | Drowned | 2 | Marasmus | 15 |
| Albuminuria | 1 | Dysentery | 1 | Mal-nutrition | 3 |
| Acute Necremia | 1 | Emphysema | 1 | Meningitis | 15 |
| Apoplexy | 8 | Epilepsy | 1 | " Tubercular | 8 |
| Ascites | 1 | Exhaustion | 1 | " Cerebro Spinal | 1 |
| Asphyxia | 1 | Erysipelas | 4 | Murder | 1 |
| Asthma | 1 | Endocarditis | 1 | Nervous Prostration | 1 |
| Asthenia | 6 | Embolism | 1 | Neurasthenia | 1 |
| Atrophy | 1 | Fever, Bilious | 1 | Old Age | 16 |
| Bright's Disease | 9 | " Intermittent | 4 | Paralysis | 9 |
| Burns and Scalds | 3 | " Malarial | 1 | Pneumonia | 40 |
| Capillary Bronchitis | 10 | " Puerperal | 1 | " Typhoid | 4 |
| Carbuncle | 1 | " Scarlet | 28 | Premature Birth | 9 |
| Cancer, Face | 3 | " Typhoid | 6 | Pyæmia | 1 |
| " Breast | 1 | Fracture, Femur | 1 | Pulmonary Apoplexy | 1 |
| " Liver | 1 | " Leg | 1 | Rheumatism, Inflammatory | 3 |
| " Lower Jaw | 1 | " Fibia and Tibula | 1 | " Heart | 1 |
| " Osteo Sarcoma | 1 | Gangrene, Lungs | 1 | Recto Vaginal Fistula | 1 |
| " Throat | 1 | Gastro Enteritis | 3 | Scrofula | 3 |
| " Uterus | 5 | General Decay of Vital Pow- | | Syncope | 1 |
| " Stomach | 2 | ers | 1 | Softening of Brain | 1 |
| " Ventricular | 1 | Hæmorrhage of Lungs | 2 | Septicæmia | 1 |
| Casualties | 1 | " Brain | 1 | Suicide | 3 |
| Cirrhosis of Liver | 2 | Hydrocephalus | 4 | Syphilis | 2 |
| Congestion of Brain | 3 | Hæmoptysis | 2 | Trismus Nascentium | 4 |
| " Laryngeal | 1 | Inanition | 8 | Tumor, Ovarian | 1 |
| " Liver | 2 | Indigestion | 2 | " Liver | 1 |
| " Lungs | 9 | Ileus | 1 | Traumatis, Spinal | 1 |
| " Kidneys | 1 | Inflammation of Brain | 2 | Inflammation | 1 |
| Consumption of Lungs | 107 | " Bowels | 5 | Ulcer, Stomach | 1 |
| Convulsions | 33 | " Stomach | 4 | " Bowels | 3 |
| Croup | 12 | " Bronchi | 10 | " Bladder | 1 |
| Cyanosis | 1 | " Kidneys | 2 | Uræmia | 1 |
| Dentition | 3 | " Larynx | 2 | Unknown, Infantile | 1 |
| Diabetes | 1 | " Liver | 2 | Whooping Cough | 4 |
| Diarrhœa | 3 | " Peritoneum | 6 | Wounds | 2 |
| Diphtheria | 15 | " Pleura | 2 | | |
| Disease of Brain | 2 | Jaundice | 1 | | 558 |

THE DEATHS REGISTERED IN THE MONTH INCLUDE.

| | | | |
|---|---|---|---|
| Under 1 year | 132 | Bet. 30 and 40 years | 49 |
| Between 1 and 2 years | 54 | " 40 and 50 " | 35 |
| " 2 and 5 years | 37 | " 50 and 60 " | 40 |
| | —— | " 60 and 70 " | 41 |
| Under 5 years | 223 | " 70 and 80 " | 31 |
| Between 5 and 10 years | 35 | " 80 and 90 " | 21 |
| " 10 and 15 " | 10 | " 90 and 100 " | 2 |
| " 15 and 20 " | 21 | " 100 and 110 " | 2 |
| " 20 and 30 " | 47 | Above 110 years | 1 |

NATIVITY, ETC.

| | |
|---|---|
| United States, White | 328 |
| Colored | 143 |
| Foreign | 87 |
| | 558 |
| Still Birth | 48 |

Among the decedents were 62 above the age of 70 years, whose combined ages were 4889 years, averaging 78 years, 10 months.

Estimated Population, 393,796. White, 338,384. Colored, 55,412. Annual Death Rate, whole population, 17.0. White, 14.73. Colored, 31.2. Births Reported, 589.

By Order: A. R. CARTER, Secretary.

JAMES A. STEUART, M. D., Commissioner of Health and Registrar.

U. S. SIGNAL OFFICE, BALTIMORE, MD., May 1, 1880.

### METEOROLOGICAL SUMMARY FOR THE MONTH OF APRIL, 1880.

PRESSURE.

Mean Monthly Barometer . . . . . 30.042 inches.
" " " . . . for past 10 years 29.980 inches.
Highest Barometer . . . . . . 30.412 inches, on the 28th.
Lowest " . . . . . . 29.559 inches, on the 10th.
Monthly Range . . . . . . 0.853 inches.

TEMPERATURE.

Mean Monthly Thermometer . . . . . 55.4 degrees.
" " " . . . for past 10 years 53.5 degrees.
Highest Thermometer, 80 degrees, on the 14th. Monthly Range . . 50 degrees.
Lowest " 30 " " 12th. Mean Daily Range . . 19.7 "

WIND, ETC.

Prevailing Direction of Wind, Northwest. Total Amount of Rainfall, 3.07 inches.
Mean Velocity of Wind, 7.5 miles per hour. Greatest Daily Rainfall 1.64 inches, on 29th.
Highest Velocity of wind, 27 m. p. h., N.W., on 29th. No. of Days on which Rain Fell, 14.
Total Movement of Wind, 5398 miles. ROBERT SEYBOTH, Sgt. Signal Corps U. S. A.

